# The Possibilities of Laparotomy

**Published:** 1890-03

**Authors:** 


### Selections and Abstracts.

### THE POSSIBILITIES OF LAPAROTOMY.

It will certainly be an encouragement to laparotomists to learn what can be done with the intestines without adding danger to the operation by sudden shock. A report comes to us from California of the following remarkable instance of vitality. A maniac performed hara-kiri in the presence of his companion, who was wakened from sleep by the noise. Standing in the middle of the floor was the suicide, with protruding intestines. Policemen were summoned, and thus the account runs: "The man was still in the middle of the floor, standing in a lake of blood, tearing out his vitals, and cutting them off in sections and throwing them in a heap on the floor.

"The officers grappled with the maniac, but notwithstanding his frightful loss of blood, he still possessed the strength of half a dozen men.

"After a desperate struggle, however, they succeeded in overpowering him. He was then handcuffed and taken to the Receiving Hospital. A great pile of intestines were left stringing about the room. When he was brougnt into the hospital three or four feet of his bloody vitals dragged upon the floor."

Nothing further in the shape of an operation could be done after getting the patient to the hospital, save trimming off a few of the remaining intestines to make him more comfortable. But he died two hours afterward.—*Medical Record.*

